# Multi-omic characterization of genome-wide abnormal DNA methylation reveals diagnostic and prognostic markers for esophageal squamous-cell carcinoma

**DOI:** 10.1038/s41392-022-00873-8

**Published:** 2022-02-25

**Authors:** Yiyi Xi, Yuan Lin, Wenjia Guo, Xinyu Wang, Hengqiang Zhao, Chuanwang Miao, Weiling Liu, Yachen Liu, Tianyuan Liu, Yingying Luo, Wenyi Fan, Ai Lin, Yamei Chen, Yanxia Sun, Yulin Ma, Xiangjie Niu, Ce Zhong, Wen Tan, Meng Zhou, Jianzhong Su, Chen Wu, Dongxin Lin

**Affiliations:** 1 Department of Etiology and Carcinogenesis, National Cancer Center/National Clinical Research Center/Cancer Hospital, Chinese Academy of Medical Sciences and Peking Union Medical College, Beijing, 100021 China; 2 Beijing Advanced Innovation Center for Genomics, Biomedical Pioneering Innovation Center, Peking University, Beijing, 100871 China; 3 Cancer Institute, Affiliated Cancer Hospital of Xinjiang Medical University, Urumqi, 830000 China; 4 School of Biomedical Engineering, School of Ophthalmology and Optometry and Eye Hospital, Wenzhou Medical University, Wenzhou, 325011 China; 5 Wenzhou Institute, University of Chinese Academy of Sciences, Wenzhou, 325000 China; 6 Jiangsu Collaborative Innovation Center for Cancer Personalized Medicine, Nanjing Medical University, Nanjing, 211166 China; 7 CAMS Key Laboratory of Genetics and Genomic Biology, Chinese Academy of Medical Sciences and Peking Union Medical College, Beijing, 100730 China; 8 Sun Yat-sen University Cancer Center, State Key Laboratory of Oncology in South China, Guangzhou, 510060 China

**Keywords:** Gastrointestinal cancer, Tumour biomarkers, Epigenetics

## Abstract

This study investigates aberrant DNA methylations as potential diagnosis and prognosis markers for esophageal squamous-cell carcinoma (ESCC), which if diagnosed at advanced stages has <30% five-year survival rate. Comparing genome-wide methylation sites of 91 ESCC and matched adjacent normal tissues, we identified 35,577 differentially methylated CpG sites (DMCs) and characterized their distribution patterns. Integrating whole-genome DNA and RNA-sequencing data of the same samples, we found multiple dysregulated transcription factors and ESCC-specific genomic correlates of identified DMCs. Using featured DMCs, we developed a 12-marker diagnostic panel with high accuracy in our dataset and the TCGA ESCC dataset, and a 4-marker prognostic panel distinguishing high-risk patients. In-vitro experiments validated the functions of 4 marker host genes. Together these results provide additional evidence for the important roles of aberrant DNA methylations in ESCC development and progression. Our DMC-based diagnostic and prognostic panels have potential values for clinical care of ESCC, laying foundations for developing targeted methylation assays for future non-invasive cancer detection methods.

## Introduction

Esophageal squamous-cell carcinoma (ESCC) accounts for 80% of esophageal cancer cases worldwide^1^ and has a 5-year survival rate of <30%.^2,3^ About 350,000 people die of ESCC every year in China where this malignancy largely occurs.^4^ Treating this disease at early stages generally results in better prognosis than at late stages, but effective biomarkers that aid early detection and/or accurate prognosis prediction are currently lacking. Aberrant DNA methylation^5,6^ plays an important role in cancer initiation and progression^7–9^ and have been investigated to derive diagnostic/prognostic biomarkers for several types of human cancer including ESCC.^10–13^ For cancer detection, differentially methylated CpG sites (DMCs) are considered better than other genetic features due to their tissue-of-origin and cancer-type specificity, early emergence during carcinogenesis and relative stability in fixed samples and body fluid over time.^14–17^ A recent clinical study has demonstrated the superiority of DMC markers when working with circulating cell-free tumor DNAs (cfDNAs).^18^ In pursuit of potential DMC-based markers, it is crucial to conduct unbiased genome-wide screening in a large number of samples. Equally important is subsequent association testing with the same patient’s other relevant genomic or transcriptomic features particularly the gene expression profile. Epigenetic anomalies often disturb gene regulation; systematically investigating the interactions between these two omics layers would help pinpoint biologically sound DMC markers. However, few previous studies have adequately fulfilled these two steps. Early works usually focused on a small number of aberrantly methylated genes instead of performing genome-wide search.^19,20^ More recent studies either ignored the genomic and transcriptomic contexts or investigated them in a different set of patients, likely due to a lack of matched multi-omics data. For example, Wang et al. interrogated the methylome of 84 TCGA ESCC patients and developed diagnostic models, but gene expression data were not consulted.^21,22^ Talukdar et al. developed a diagnostic 7-CpG panel based on methylation profiling of more than 100 ESCC samples collected from Africa, Asia and South America countries, but they weighted each CpG marker based on gene expression from TCGA ESCC patients (mostly Caucasian).^13^ Chen et al. integrated DNA methylation and gene expression profiles from the same samples, but the sample size was too small (*n* = 4).^23^ Furthermore, although DMC sites in cancer genomes bear ethnic specificity,^24^ Caucasian samples are by far the mostly used for biomarker discovery, with only a couple of exceptions.^13,23^ No large-scale methylome interrogation has been carried out for Chinese ESCC patients.

In our previous study,^25^ we have performed whole-genome sequencing and RNA-sequencing on 91 Chinese ESCC patients’ matched tumor and adjacent normal tissue samples. In the present study, we continued to profile genome-wide DNA methylation of the same samples and correlate DMCs with a variety of gene expression alterations as well as somatic and germline variants specific to ESCC. Based on the results, we have developed a diagnostic model comprised of 12 promoter/gene-body DNA methylation CpG sites that robustly distinguishes ESCC from adjacent tissues or normal esophagus in multiple patient sets. We have also developed a prognostic model comprised of 4 promoter/gene-body CpG sites that can classify ESCC patients into high-risk and low-risk groups. The host genes of identified markers have potentially functional roles in ESCC development and progression, as implicated in the literature or by our in-vitro experiments. Overall, this study demonstrates that the ESCC genome abounds with specific DNA methylation patterns that could be effective diagnostic or prognostic biomarkers and potential mediators of tumor development and/or progression.

## Results

### Overview of differentially methylated CpG sites in ESCC

**Fig. 1:**
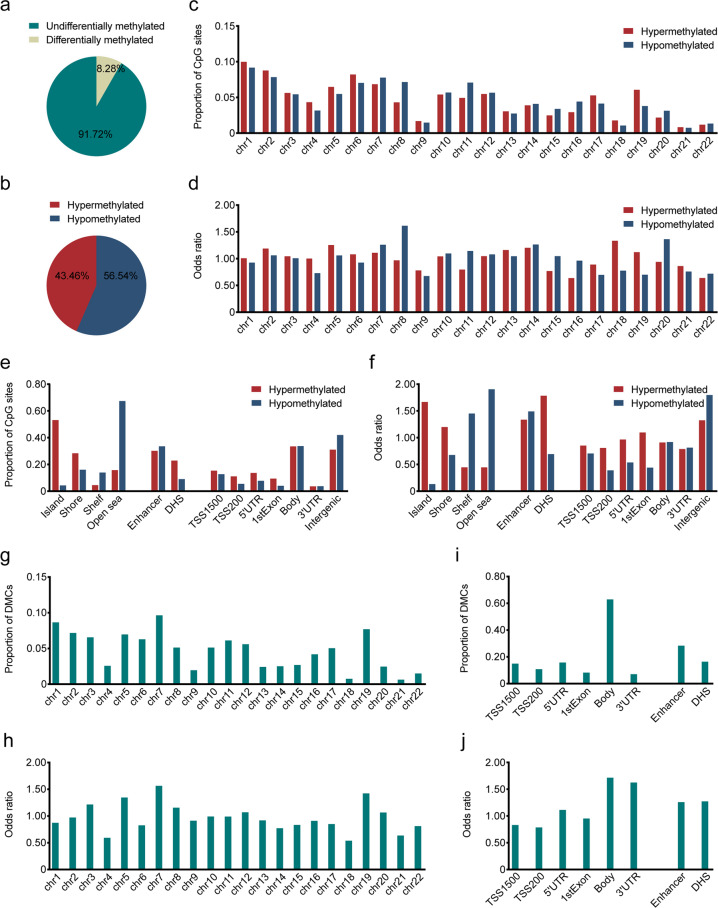
Characteristics of differentially methylated probes in ESCC. **a** The proportion of all filtrated CpG sites that are differentially methylated or not methylated. **b** The proportion of differentially hyper-methylated (red) or hypo-methylated (blue) CpG islands. **c**, **d** The proportion (**c**) and odds ratio (**d**) of hyper-methylated (red) or hypo-methylated (blue) CpG sites in different chromosomes. **e**, **f** The category (**e**) and odds ratio (**f**) of genomic locations for hyper-methylated (red) or hypo-methylated (blue) sites. **g**, **h** The proportions (**g**) and odds ratio (**h**) of methylated CpG sites correlated with the expression levels of genes in different chromosomes. **i**, **j** The category (**i**) and odds ratio (**j**) of genomic locations for CpG sites correlated with genes expression. Odds ratio was computed against the general distribution and *P* value was computed by Hypergeometric test. Island, CpG island; shore, 0–2 kb from CpG island; shelf, 2–4 kb from CpG island; open sea, other genomic regions; TSS1500, 200–1500 bases upstream of the transcriptional start site (TSS); TSS200, 0–200 bases upstream of the TSS; 5’UTR, within the 5’ untranslated region and between the TSS and the ATG start site; body, between the ATG and stop codon regardless the presence of introns, exons, TSS or promoters; 3’UTR, between the stop codon and poly A signal

Among 429,717 probes (out of 467,079, 92%) that had passed quality control, 35,577 (8.28%) were differentially methylated between tumor and adjacent normal samples (FDR *q* < 0.05, absolute median methylation difference |MMD | > 0.20; Fig. 1a), with 56.54% (20,114/35,577) of these DMCs hypo-methylated (Fig. 1b). The distribution of DMCs varied among chromosomes (Supplementary Fig. S1a, b), mostly enriched in Chromosome 8 (odds ratio (OR) = 1.32, *P* = 1.00e-89) and mostly absent in Chromosome 22 (OR = 0.67, *P* = 1.00e-45). Hyper-methylated sites were mostly enriched in Chromosomes 18 and 19 (OR = 1.32, *P* = 3.60e-6, OR = 1.11, *P* = 4.25e-4) whereas hypo-methylated sites in Chromosome 8 (OR = 1.60, *P* = 2.24e-68), respectively (Fig. 1c, d). Furthermore, DMCs were significantly enriched in intergenic and enhancer regions (Supplementary Fig. S1c, d), with more hypo- than hyper-methylated CpG sites in intergenic regions and similarly abundant hyper- and hypo-methylated sites in enhancer regions (Fig. 1e, f). Hyper-methylated CpG sites were also enriched in CpG islands (OR = 1.66, *P* = 1.00e-1502) and DNase I hypersensitivity sites (OR = 1.77, *P* = 6.06e-258), while hypo-methylated sites also enriched in open sea (OR = 1.89, *P* = 1.00e-4373) (Fig. 1e, f). We found more hyper- than hypo-methylated sites within promoter regions (Fig. 1e, f). At the chromosome level, Chromosome 8 was enriched with hypo-methylated sites mainly found in open sea, intergenic and enhancer regions; Chromosomes 18 and 19 were enriched with hyper-methylated sites mainly found in CpG islands, promoter and DNase I hypersensitivity sites (Supplementary Fig. S1e−j).

**Fig. 2:**
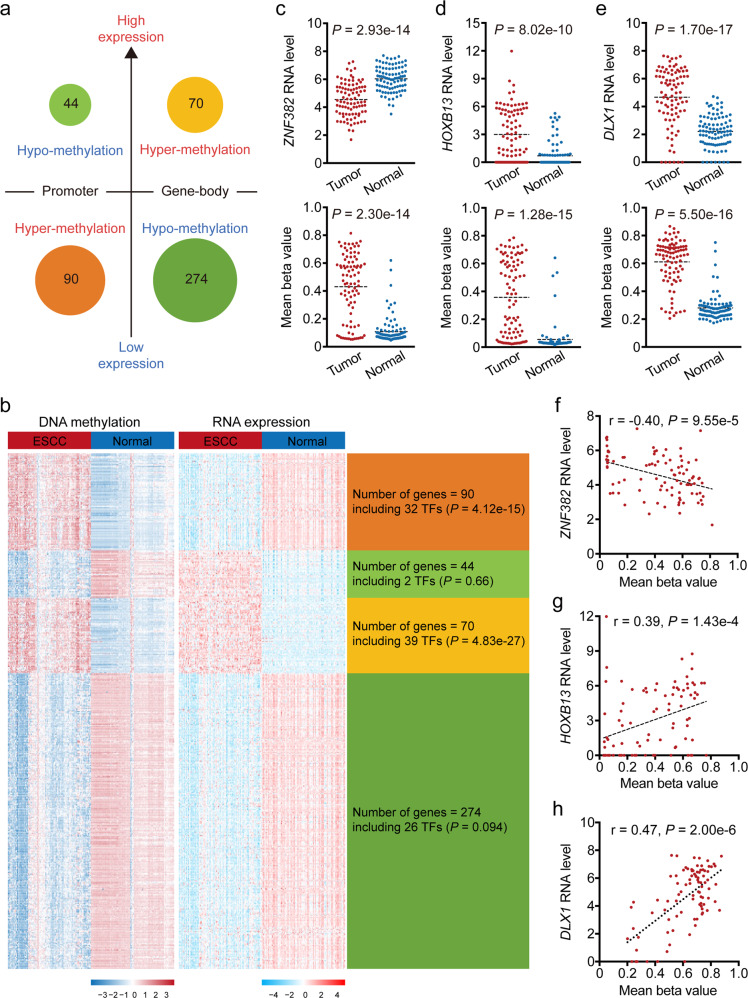
Integrative analysis of whole-genome DNA and RNA-sequencing data uncovered methylation-mediated dysregulation of multiple TFs in ESCC. **a**, **b** The association between promoter or gene-body methylation and host gene expression were identified. There are four clusters: genes (*n* = 90) that are hyper-methylated in promoter with low expression in ESCC; genes (*n* = 44) that are hypo-methylated in promoter with high expression; genes (*n* = 70): that are hyper-methylated in gene-body with high expression; genes (*n* = 274) that are hypo-methylated in gene-body with low expression. Number of known TFs are shown in each cluster. *P* value was computed by Hypergeometric test. **c** Differential expression (top) and promoter methylation (bottom) levels of *ZNF382* in ESCC and normal samples. **d**, **e** Differential expression (top) and gene-body methylation (bottom) levels of *HOXB13* (**d**) and *DLX1* (**e**) in ESCC and normal samples. **f** The correlation between mRNA expression and promoter DNA methylation levels of *ZNF382*. **g**, **h** The correlation between mRNA expression and gene-body DNA methylation levels of *HOXB13* (**g**) and *DLX1* (**h**). *P* of Student’s *t* test for gene expression and Wilcoxon signed-rank test for methylation. Genes mRNA expression level (RSEM) was added by 1 and then log2 transformed. Dotted short line indicates mean expression level of each gene

Commercial DNA methylation arrays are intentionally focused on DNA methylation CpGs at promoters and gene bodies, which often regulate the expression of host genes in a cis manner. Even so, the genome of our ESCC samples still contained more-than-expected hyper-methylation in promoter and adjacent regions, while hypo-methylation dominates genome wide, as observed in several other cancer types.^7,9,17^ The methylation status of 3241 (9.11%) promoter or gene-body DMCs were significantly correlated with the expression levels of their host genes in ESCC, quantified using RNA sequencing data (|Spearman’s correlation coefficient *r* | > 0.30, FDR *q* < 0.05). These DMCs were mostly overrepresented in Chromosome 7 (OR = 1.55, *P* = 1.80e-14; Fig. 1g, h) and were more likely to reside at gene-bodies than at promoter and adjacent regions (OR = 1.70, *P* = 6.22e-197; Fig. 1i, j). Because DMCs at promoters and gene bodies often affect host gene expression differently,^26^ we investigated promoter-DMC involved negative expression-methylation correlations and gene-body-DMC involved positive expression-methylation correlations, using same patients’ gene expression profiles obtained in our previous study^25^ (Fig. 2a, b). Among protein coding genes differentially expressed between tumor and adjacent normal samples, 90 downregulated and 44 upregulated genes were associated with 224 hyper-methylated and 70 hypo-methylated CpG sites at their promoter regions, respectively; 274 downregulated and 70 upregulated genes were associated with 764 hypo-methylated and 221 hyper-methylated CpG sites in their gene-bodies, respectively (Fig. 2a, b). We then looked into the methylation-expression correlation in Chromosomes 8, 18, 19. Chromosomes 8 mainly contained genes whose expression levels were associated with hypo-methylated sites in gene bodies (Supplementary Fig. S2a, b); Chromosome 19 mainly contained genes whose expression levels were associated with promoter hyper-methylated sites (Supplementary Fig. S2c, d). No preference was observed in Chromosome 18.

Host genes potentially dysregulated by negatively correlated promoter-DMCs were enriched in the GO categories of metal ion binding, transcription factor activity and transcription regulation, whereas host genes potentially dysregulated by positively correlated gene-body DMCs were enriched in the GO categories of system development and cell part morphogenesis (Supplementary Fig. S3). In light of this result, we examined the overlap between these DMC-associated genes with known human transcription factors (TFs)^27^ and found significant TF enrichment in hyper-methylation associated genes, including 32 of 90 downregulated genes (*P* = 4.12e-15) associated with promoter hyper-methylation and 39 of 70 upregulated genes (*P* = 4.83e-27) associated with gene-body hyper-methylation (Fig. 2b). The former group (32 downregulated TFs) were mostly the members of the zinc finger gene family, such as *ZNF382*^28^ (Fig. 2c, f), whereas the latter group (39 upregulated TFs) included 29 potential oncogenic Homeobox genes such as *HOXB13* and *DLX1*^29^ (Fig. 2d, e, g, h), suggesting genome-wide DNA methylation anomalies may have led to the dysregulation of multiple TFs involved in a variety of molecular processes contributing to ESCC initiation and progression. Twenty-three of the 32 downregulated TFs (71.88%) locate in Chromosome 19, accounting for 85.19% (23/27) of all the protein coding genes in that chromosome that were associated with hyper-methylated promoter CpGs and downregulated in tumor samples (Supplementary Fig. S2d).

### Differentially methylated CpG sites are associated with ESCC-specific genetic variations

**Fig. 3:**
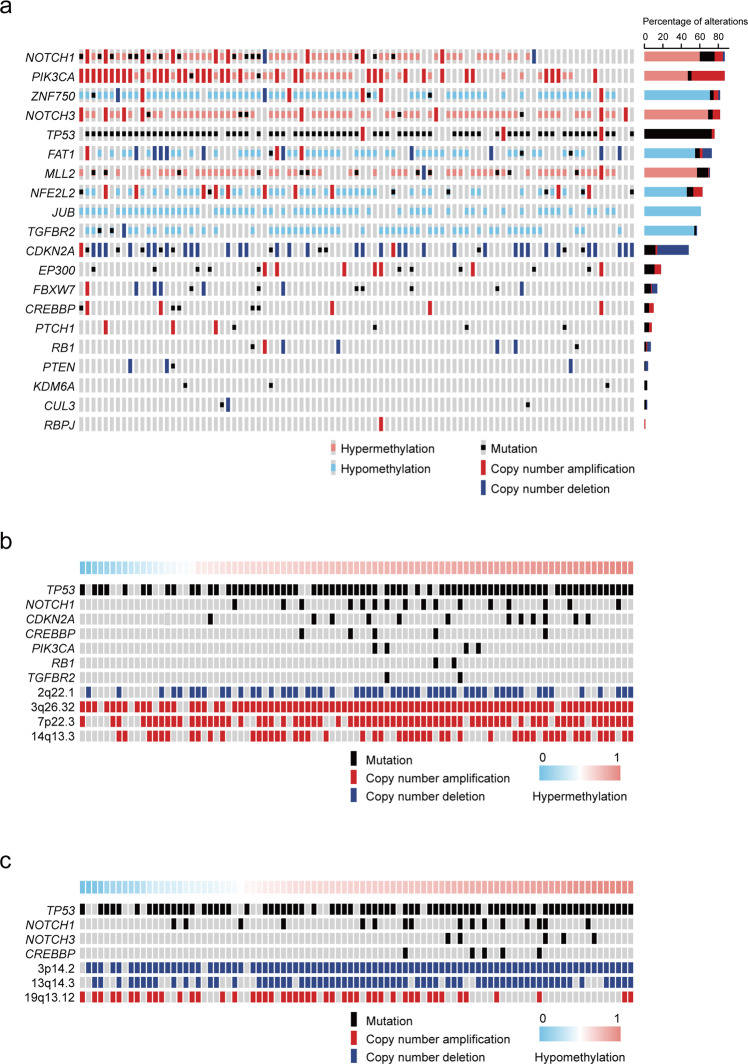
Hyper- and hypo-methylation events across ESCC and integrated profiling of ESCC driver genes. **a** Map overview of genetic and epigenetic alterations in 20 ESCC driver genes previously identified. ach column denotes an individual patient and each row represents the status of one gene including somatic mutations (black squares), copy number amplifications (red bars), copy number deletions (blue bars), hyper- (pink bars) and hypo-methylated events (azure bars). Wild-type cases are in gray. Right, percentage of alterations for each gene in 91 ESCC patients while the *X* axis represents total percentage of alterations for each gene. **b**, **c** We tested recurrent genetic alterations in ESCC for their associations with frequency of hyper- (**b**) or hypo-methylated event (**c**). Significant associations (Wilcoxon *P* < 0.05) were shown in above and labeled by gene symbol for somatic mutations or cytoband for amplifications and deletions. Each column denotes an individual patient and each row is one genetic alteration including somatic mutations (black bars), copy number amplifications (red bars) and copy number deletions (blue bars). Wild-type cases are in gray. Top color bars represent the frequency of DNA methylation

We found recurrent promoter or gene-body DMCs (each in >51% (46/91) patients) in 9 previously identified ESCC driver genes *FAT1*, *NOTCH1*, *JUB*, *MLL2*, *PIK3CA*, *TGFBR2*, *NFE2L2*, *NOTCH3* and *ZNF750*^25^ (Fig. 3a; Supplementary Data S1). As many as 97% (88/91) of our patients had promoter or gene-body DMCs in these genes. Surprisingly, no DMCs were found in *TP53*. Among DMC-correlated TF genes, some have been implicated in the progression of ESCC, including zinc finger genes such as *ZNF382*, *ZNF582* and *ZNF667*^28,30,31^ and Homeobox genes such as *BARX1*, *HOXA13* and *HOXC10.*^32–34^ All 7 CpG sites at the promoter of *ZNF382*, a NF-κB inhibitor frequently downregulated in ESCC,^28,35^ were recurrently hyper-methylated in our data: at least one of the 7 DMCs were found in 75.82% (69/91) ESCC genomes. We also found hypo-methylation in the promoter of *TP63*, which is part of a core regulatory circuitry for ESCC.^36^

The frequencies of differential methylation events were then correlated with recurrent ESCC genomic variants that we have previously identified.^25^ We found that hyper-methylation events were significantly correlated with somatic mutations in the genes *RB1*, *NOTCH1*, *CDKN2A* and *PIK3CA*, 3q26.32, 7p22.3 and 14q13.3 amplifications and 2q22.1 deletions; hypo-methylation events were significantly correlated with somatic mutations in the genes *CREBBP* and *NOTCH3*, 19q13.12 amplifications and 3p14.2 and 13q14.3 deletions (Fig. 3b, c and Supplementary Data S2, 3). *Cis*-meQTL analysis indicated 292 DMCs associated with 4864 nearby SNPs (within a 100-kb window centering each DMC) in ESCC tissues and 2064 DMCs associated with 29,321 SNPs in adjacent normal tissues (Supplementary Data S4, 5 and Supplementary Fig. S4). Compared with adjacent normal, 1974 DMCs lost genetic control in tumor genomes and 202 DMCs gained new correlations. Moreover, ESCC-associated SNPs (14,761, nominal *P* < 0.05) ascertained from our previous GWAS studies^37^ were enriched in the identified meQTLs (tumor, *P* = 5.66e-260; normal, *P* = 1.00e-1240), which suggests that perturbed DNA methylation may contribute to ESCC predisposition.

### Differentially methylated CpG sites are effective diagnostic/prognostic markers for ESCC

**Fig. 4:**
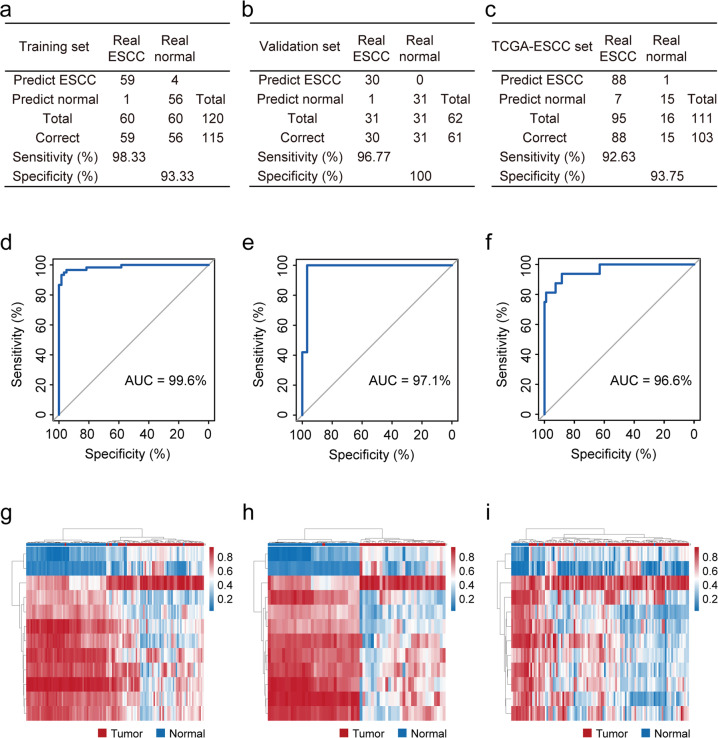
Diagnosis of ESCC with a DNA methylation panel. **a**–**c** The confusion tables of binary results of diagnostic prediction model in the training (**a**), validation (**b**) and TCGA ESCC (**c**) datasets. **d**–**f** The receiver operating characteristic curve (ROC) of the diagnostic prediction model in the training (**d**), validation (**e**) and TCGA ESCC (**f**) datasets. **g−i** Unsupervised hierarchical clustering and heatmap of 12 methylation markers screened for constructing the diagnostic prediction model in the training (**g**), validation (**h**) and TCGA ESCC (**i**) datasets

Not only were the identified DMCs associated with various molecular characteristics of ESCC, together they could distinguish ESCC from normal esophageal tissues (Supplementary Fig. S5a). We hypothesized that a relatively small number of DMC markers are sufficient for ESCC diagnosis. To identify such markers, we randomly divided the patients into a training set (*n* = 60) and a validation set (*n* = 31) and started with the 1034 promoter/gene-body DMCs whose methylation levels were negatively (or positively) correlated with the expression levels of their host genes. Tumors and adjacent normal samples in the training set were adequately separated by the 1034 DMCs (Supplementary Fig. S5b). Applying random-forest and LASSO to these DMCs generated a model of 12 DMCs (Supplementary Table S1). This model achieved 98.33% sensitivity and 93.33% specificity in the training set (Fig. 4a) and 96.77% sensitivity and 100% specificity in the validation set (Fig. 4b). We also computed receiver operating characteristic (ROC) curves and the area-under-curve values (AUCs) were 99.6% and 97.1% in the training and the validation sets, respectively (Fig. 4d, e). When tested in other ESCC datasets, including TCGA ESCC data and additional GEO datasets (GSE52826 and GSE77991), this diagnostic model consistently showed high sensitivity, specificity and AUCs (Fig. 4c, f and Supplementary Fig. S6a−d), which indicates robustness and generalizability. Unsupervised hierarchical clustering based on these DMCs clearly distinguishes ESCC from normal tissue samples (Fig. 4g−i).

**Fig. 5:**
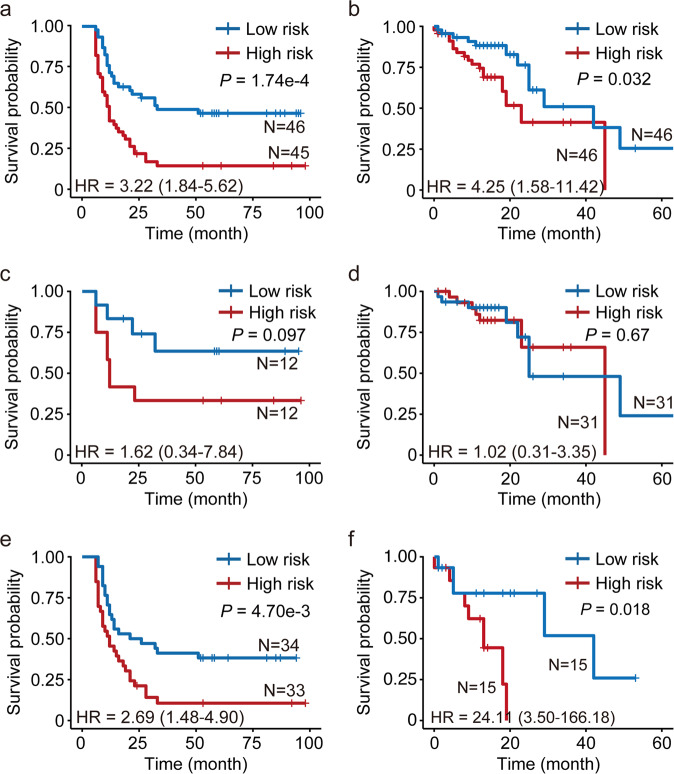
The correlation of the methylation signature and survival time in patients with ESCC. **a**, **b** Kaplan–Meier survival curves for all our patient sample (**a**) and all TCGA ESCC patient sample (**b**). **c**, **d** Kaplan–Meier survival curves for patients with early stage ESCC in our sample (**c**) and in TCGA ESCC sample (**d**). **e**, **f** Kaplan–Meier survival curves for patients with advanced stage ESCC in our sample (**e**) and in TCGA ESCC sample (**f**). High- or low-risk group was defined by the weighted hazard ratios of the 4 methylation sites in patients. The *P* value was calculated by log rank test. HR and 95% CI was computed with Cox hazard proportion model

We looked for potential prognosis markers among DMCs based on how strong they were associated with the overall survival (OS) time of ESCC patients in our sample and the TCGA ESCC sample. For each DMC, we constructed a Cox regression model including that DMC as a single predictor and age, sex, smoking status, drinking status and tumor TNM stage as covariates. Four DMCs (cg23378365, cg06090867 and cg03244277 in the promoters of *CYFIP2*, *UBXN10*, *AREG*, respectively, and cg02370667 in the gene-body of *NECAB2*) were significantly associated with patient survival in our sample. We then constructed a prognostic model by summing the methylation levels of these 4 DMCs, each weighted by the hazard ratio (HR) in the corresponding Cox regression result (Supplementary Table S2). This model classified our patients as having high or low prognostic risk (Supplementary Table S3) where the high-risk patients had significantly shorter median OS than others (12 versus 33 months, *P*_log-rank_ = 1.74e-4; Fig. 5a), the HR being 3.22 (95% confidence interval (CI) = 1.84−5.62) adjusted for age, sex, smoking status, drinking status and tumor TNM stage. Applying this model to the TCGA ESCC sample yielded a similar result: the predicted high-risk patients had significantly shorter median OS than low-risk patients (23 versus 42 months, *P*_log-rank_ = 0.032; Fig. 5b), the HR being 4.25 (95% CI = 1.58−11.42) adjusted for age, sex and tumor TNM stage.

We further carried out survival analysis in patients with different tumor stages to evaluate the discriminating ability of our prognostic panel. Within early-stage (I and II) patients in our sample, the low-risk group had longer OS time than the high-risk group (Fig. 5c), although the statistic was marginally significant (*P*_log-rank_ = 0.097; HR = 1.62, 95% CI 0.34−7.84), probably due to relatively small sample size (*n* = 24). Our model did not perform as well in early-stage patients of the TCGA ESCC cohort (Fig. 5d). For patients with advanced disease (stage III and IV), our model was particularly strong. In our sample, the median OS time in advanced ESCC patients was 12 months for the high-risk group versus 23.5 months for the low-risk group (*P*_log-rank_ = 4.70e-3; HR = 2.69, 95% CI 1.48−4.90; Fig. 5e). Similarly, in the TCGA ESCC sample, the median OS time in advanced ESCC patients was 13 and 42 months for high and low-risk groups, respectively (*P*_log-rank_ = 0.018; HR of 24.11, 95% CI 3.50−166.18; Fig. 5f).

We examined the differential methylation status of the above 16 diagnostic/prognostic markers in the TCGA ESCC data. Ten of them showed similarly significant methylation changes in TCGA ESCC samples compared with normal samples. The rest six markers (cg05446471, cg19310604, cg21041579, cg03244277, cg06090867, cg23378365 in *HDAC11*, *HOXC10*, *SYNE3*, *AREG*, *UBXN10* and *CYFIP2*, respectively) showed similarly significant yet less intensive methylation changes (*P* < 0.05, absolute methylation difference <0.20). We also compared the methylation patterns of the 16 markers across 22 cancer types that have available 450 K array data and normal samples in the TCGA database, with esophageal cancer samples further divided into ESCC and esophageal adenocarcinoma (EAC). The result (Supplementary Fig. S7) confirmed that these markers are ESCC specific.

### Functional implications of identified diagnostic and prognostic markers

Some DMC markers we identified locate in the promoters or gene bodies of protein-coding genes and may have contributed to ESCC development or progression by affecting the expression of these genes. To test our hypothesis, we first looked for markers whose methylation levels were correlated with the expression levels of host or nearby genes. Among the 12 DMC markers for ESCC diagnosis, cg10085326, cg24276395, cg05446471, cg21553182 reside at the promoters of *MMP13*, *YEATS2*, *HDAC11* and *ZNF578*, respectively. We classified patients into two groups by the median methylation levels of each site and then compared the expression levels of the corresponding genes. Patients with high methylation of each marker had significantly lower expression levels of these 4 genes in ESCC than patients with low methylation (Supplementary Fig. S8a−d). The methylation status of each marker was negatively correlated with the expression level of the corresponding host gene (all Spearman *r* < –0.30, *P* < 0.05, Supplementary Fig. S9a−d).

The other 8 diagnostic DMCs locate in the gene-body of *AFF3*, *PDE4D*, *SYNE3*, *SLC8A3*, *CPS1*, *HOXC10*, *LDB2* and *PACRG*, respectively. The high methylation status in the 8 sites corresponded to significantly higher expression levels than low methylation status, except for *AFF3* (Supplementary Fig. S8e−l). The expression levels of these genes were positively correlated with the methylation levels in their gene bodies (all Spearman *r* > 0.30, *P* < 0.05, Supplementary Fig. S9e−l).

Three of the four DMCs associated with the survival time in ESCC patients, cg23378365, cg06090867, cg03244277 locate at the promoters of *CYFIP2*, *UBXN10* and *AREG*, respectively; cg02370667 resides in the gene-body of *NECAB2*. The expression levels of these genes showed no significant difference between high and low methylation groups of each marker except for *NECAB2* (Supplementary Fig. S8m−p). The expression levels of *NECAB2* and *UBXN10* were significantly correlated with the methylation levels of corresponding CpG sites (*NECAB2*, *r* = 0.42, *P* = 3.48e-5, Supplementary Fig. S9m; *UBXN10*, *r* = 0.22, *P* = 0.033, Supplementary Fig. S9o).

**Fig. 6:**
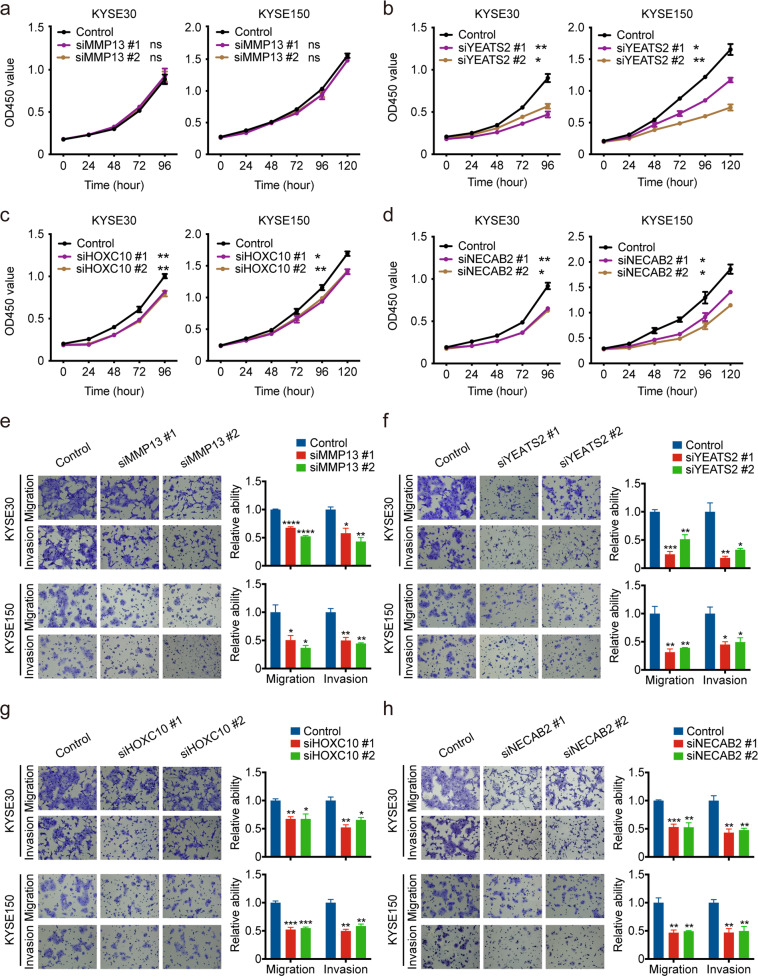
Effects of silencing some genes in diagnostic and prognostic panels on ESCC cell phenotypes. **a**–**d** Silencing the expression of *MMP13* (**a**), *YEATS2* (**b**), *HOXC10* (**c**) and *NECAB2* (**d**) significantly suppressed KYSE30 and KYSE150 cell proliferation. **e**–**h** Silencing the expression of *MMP13* (**e**), *YEATS2* (**f**), *HOXC10* (**g**) and *NECAB2* (**h**) significantly suppressed KYSE30 and KYSE150 cell migration and invasion. Left panel shows representative cell migration and invasion images and right panel shows quantification statistics. Data represent mean ± SEM from 3 independent experiments. *P < 0.05; **P < 0.01; ***P < 1.00e-3; ****P < 1.00e-4 and ns not significant of Student’s *t* test compared with corresponding control

Next, we focused on marker host genes whose expression levels significantly increased in ESCC samples and associated with the methylation levels of corresponding DMC markers, hypothesizing that knocking down the expression of such genes would diminish the malignancy of ESCC cells in vitro. *MMP13*, *YEATS2* and *HOXC10* with DMC markers in their promoters and *NECAB2* with a gene-body DMC marker were selected for the functional experiments. These four genes were overexpressed in our ESCC samples compared to matched normal tissue samples and the expression levels were strongly correlated with the methylation levels of corresponding DMCs (|Spearman’s *r* | > 0.30). We knocked down the expression of these genes in ESCC cell lines, one at a time (Supplementary Fig. S10). Silencing the expression of *YEATS2*, *HOXC10* or *NECAB2* by siRNA significantly inhibited ESCC cell proliferation, migration and invasion; silencing the expression of *MMP13* significantly suppressed ESCC cell migration and invasion but not proliferation (Fig. 6a−h).

## Discussion

Despite the promising future of targeted DNA methylation assays in ESCC detection, only recently have we begun to rely on large sample size and genome-wide profiling, and the interactions between DNA methylation and other omic features (e.g., aberrant gene expression and genomic alterations) are too often ignored.^23,38–44^ A large-scale, systematic screening of diagnostic and prognostic DMC markers has not been conducted on Chinese samples before, even though China has the highest incidence of ESCC around the world. Here, we explored genome-wide DNA methylation anomalies of 91 Chinese ESCC patients. By comparing their tumor and paired normal samples, we identified 35,577 DMCs and characterized their genome-wide distribution patterns. By further integrating genomic and gene expression data of the same samples, we associated many of these DMCs with ESCC-specific genomic or transcriptomic variations, such as somatic mutations in putative ESCC driver genes, germline SNPs associated with ESCC predisposition, as well as aberrant expression of characteristic functional gene sets and pathways.

Notably, the expression of multiple TFs, including several members of the zinc finger family and the homeobox family, were perturbed in ESCC likely due to associated DMCs. Two of these DMCs, associated with *ZNF578* and *HOXC10* respectively, were selected into our diagnostic panel and we validated the functional role of *HOXC10* in vitro. Previous pan-cancer analyses have shown that DNA methylation plays a predominant role in dysregulating TFs in general and the homeobox family in particular.^29,45^ Since TFs are “master regulators” of biological processes and pathways critical for the development and differentiation of specific cell types^46^ and their defining functions,^47^ DNA methylation associated with TF dysregulation could happen at an early or even precancerous stage of ESCC, making them good candidates for early diagnosis markers. In our data, most of the perturbed TFs locate in Chromosome 19, suggesting that Chromosome 19 targeted marker detection could be an alternative to whole-genome screening when the latter is too expensive (e.g., for a very large cohort).

Based on featured DMCs, we developed a panel of 12 methylation CpG sites that can well distinguish ESCC tumor from normal tissues and a linear model of 4 CpG sites that can classify patients into different risk groups in terms of overall survival time. Both models were validated in public ESCC datasets. Recently a 7-CpG diagnostic panel for ESCC has been developed using a large non-TCGA discovery cohort.^13^ When applied to TCGA ESCC data, the model showed an AUC of 89%, lower than ours 96.6%. Without looking into the not-yet-released data of that study, we can only speculate on the reasons: (a) we processed all samples into freshly frozen tissues while they used formalin-fixed paraffin-embedded (FFPE) tissues; (b) we only included tumor samples with >75% neoplastic cells, while their filtering threshold was 50%; (c) we combined random forest and the least absolute shrinkage and selection operator (LASSO)-penalized logistic regression for marker screening, while they performed partial least square-discriminant analysis (PLS-DA); (d) we integrated gene expression data of the same patients while they used those of TCGA ESCC patients. A 9-CpG panel has been previously developed for ESCC prognosis,^38^ but was based on Illumina’s GoldenGate methylation array with only 1505 CpG sites. Only one marker in that panel showed correlation with its host gene.

Following the cis-effect hypothesis of DMCs, we investigated the host genes of the DMC markers in our models. All 12 diagnostic-marker host genes were dysregulated in ESCC samples compared with matched adjacent normal samples. Among them, *MMP13* encodes a member of the matrix metalloproteinase family that can be dysregulated in esophageal cancer.^48,49^ *YEATS2* encodes a scaffolding subunit of the Ada-two-A-containing (ATAC) complex implicated in lung tumorigenesis.^50^ *HDAC11* encodes a class IV histone deacetylase involved in multiple types of cancer.^51–53^ *AFF3* is a potential tumor suppressor in lung cancer.^54^ *PDE4D* and *CPS1* were dysregulated in a variety of cancer types.^55–60^ *HOXC10* has been associated with many cancer types including ESCC.^34,61–64^ The roles of *SYNE3, SLC8A3*, *LDB2* and *PACRG* in ESCC development remain unknown and thus warrant further investigations. Three out of four prognostic-marker host genes were dysregulated in our ESCC samples. *AREG* encodes the epidermal growth factor receptor (EGFR) ligand amphiregulin, an often-upregulated prognostic marker in several types of human cancer.^65–69^ The methylation of *AREG* has been associated with survival in patients with astrocytoma.^69^ The protein product of *CYFIP2* is involved in p53-dependent apoptosis induction.^70^ Decreased expression of *CYFIP2* can promote cancer cell growth in vitro^71^ and has been reported in gastric cancer.^72^ The roles of *UBXN10* and *NECAB2* in ESCC progression remain to be elucidated. We demonstrated in vitro that *MMP13*, *YEATS2*, *HOXC10* and previously unreported *NECAB2* could contribute to ESCC progression when upregulated.

We validated the 16 DMC markers in the TCGA 450 K microarray data. Although they all had similarly significant methylation changes, 6 sites, including one at the functionally validated *MMP13*, changed so little that they would not have been discovered if we had screened the TCGA data. We also examined previously reported DNA methylation markers and their host genes^13,21–23,38,39^ in our samples. Of 30 differentially methylated CpG regions/sites that involve 25 genes, six regions (in *PAX9*, *THSD4*, *TWIST1*, *EPB41L3*, *GPX3* and *COL14A1*, respectively) and 4 CpG sites (cg20655070, cg27062795 in *ZNF542* and cg04550052, cg04698114 in *SALL1*) had similarly significant methylation changes. The methylation status of one region (in *CDH5*) and 8 CpG sites was undetermined in our samples, as the 450 K microarray we used does not include corresponding probes. No significant methylation difference was detected regarding three regions (in *SIM2*, *MLH1* and *CDX1*, respectively) and 7 CpG sites (cg15830431, cg19396867, cg26671652, cg20295442, cg20912169, cg22383888, cg12973591 in *STK3*, *ZNF418*, *ADHFE1*, *EOMES* and *TFPI2*, respectively). These discrepancies may reflect ethnic divergency.

Though equipped with multi-omic data, we decided not to identify markers from other omics layers and then integrate them into current DMC-only diagnostic/prognostic panels. On the one hand, it might help explain more individual heterogeneity but not necessarily lead to more discriminating power. For example, we and others have found genomic alterations previously considered tumor-specific (e.g., driver mutations and copy number variations) in normal aging esophagus,^73^ so incorporating these genomic features may end up adding noise. On the other hand, multi-analyte tests, i.e., checking markers from different omic layers, are presumably more complicated and expensive. In a clinical setting, comprehensiveness is rarely the priority and often traded-off for cost efficiency; fewer markers are preferred if they can do the same job. Finally, as mentioned earlier, DMC markers have unique advantages over other omics markers.^74^

The current study has several limitations. First, our DNA methylation profiling is limited by the fixed design of microarrays. Sequencing-based profiling may provide more insights due to improved base-pair resolution and better genome coverage. Second, marker screening and functional validation were limited to DMCs primarily affecting their host protein-coding genes in a cis manner, while DMCs can exert influences at a distance (i.e., in a trans manner) and on non-coding elements as well. Moreover, these influences may not be strictly one-to-one but rather form an interconnected network. Since our panels perform relatively well, we speculate that they may capture some central relations within this “network,” which requires further investigation. Third, 73.63% (67/91) of the ESCC samples we used to develop the diagnostic and the prognostic models were at an advanced stage (III or IV). Although both models were validated in the TCGA ESCC set, 67.39% (62/92) of which are at an early stage (I or II), their efficacy in patients with early-stage ESCC or precancerous lesions needs additional evaluation. Lastly, the results of this study only implicate a potential functional role of DNA methylation in ESCC development and progression, which warrants further mechanistic investigations.

In conclusion, our characterization of genome-wide DNA methylation anomalies using a multi-omic approach in 91 Chinese ESCC patients has supported that aberrant DNA methylation is an important part of ESCC development and progression. This study has also targeted a small number of potentially functional methylation CpG sites able to distinguish tumors from normal tissues or classify patients into high or low-risk groups. Using these CpG sites, we have constructed DNA-methylation panels for molecular diagnosis and prognosis of ESCC and validated them in multiple public datasets. The panels are potentially useful for clinical care of ESCC and it would be interesting to evaluate their utilities on non-invasively collected, small amount of tumor DNA, such as those obtained using Cytosponge^75^ or liquid biopsy.^76^

## Materials and methods

### Study subjects and biospecimens

Individuals with ESCC (*n* = 91) were recruited from Chinese Academy of Medical Sciences Cancer Hospital (CAMSCH; Beijing, China) and Zhejiang Cancer Hospital (ZCH; Hangzhou, China) between 2010 and 2014. All subjects underwent esophagostomy and had not been treated with chemotherapy or radiotherapy prior to the surgery. ESCC tumor and adjacent normal tissue (≥5 cm from the tumor margin) were collected from each individual as described previously.^25^ Histological evaluation was conducted by two pathologists to ensure that tumor specimens contained an average of >75% tumor cell nuclei with <20% necrosis, whereas normal specimens contained no tumor cells. The demographic characteristics and clinical data of the study subjects were obtained from medical records. Written informed consent was obtained from every subject and this study was approved by the Institutional Review Board of CAMSCH and ZCH.

### Cell lines and cell culture

Human ESCC cell lines KYSE30 and KYSE150 were generous gifts from Dr Y. Shimada at the Kyoto University. These cell lines were maintained in RPMI 1640 medium supplemented with 10% fetal bovine serum (FBS). Cell lines used in this study were authenticated by short tandem repeat profiling and were free of mycoplasma infection.

### RNA interference

Small interfering RNA (siRNA) oligos targeting *MMP13*, *YEATS2*, *HOXC10* or *NECAB2* were provided by JTSBIO (Supplementary Table S4). The transfection of each siRNA was performed with Lipofectamine 3000 (Invitrogen). The specific sequences for target genes are provided in the supplementary information.

### Quantitative real-time PCR analysis

Total RNA was extracted with Trizol reagent (Invitrogen) and the reverse transcription was performed using PrimeScript^TM^ RT reagent kit (Takara). Quantitative real-time PCR (qRT-PCR) was performed in triplicate using TB Green Premix Ex Taq (Takara). The primer sequences used for qRT-PCR of interest genes are shown in Supplementary Table S4.

### Western blot analysis

Proteins were extracted using RIPA lysis buffer (Solarbio, R0020) containing PMSF (Solarbio, P0100), phosphatase inhibitor cocktail I and II (MCE, HY-K0021 and HY-K0022). In total, lysate containing 10–20 μg of protein was separated on SDS-PAGE and transferred to PVDF membranes (Millipore). Antibodies against MMP13 (ab51072), HOXC10 (ab153904) and GAPDH (ab181602) were from Abcam while antibodies against YEATS2 (24717-1-AP) and NECAB2 (12257-1-AP) were from Proteintech. The signal was captured with a SuperSignal^TM^ West Pico/Femto Chemiluminescent Substrate kit (Thermo Fisher, 34580) analyzedthrough the Amersham Imager 600.

### Cell viability and migration or invasion assays

Cell viability was measured after incubation with CCK-8 (Dojindo). Invasion assays were performed in 24-well chambers (Corning) coated with Matrigel (BD Biosciences). Cells (20 × 10^4^) in serum-free medium were added to the coated chamber and incubated for 18 or 24 h before fixed with methanol and stained with 0.5% crystal violet. Migration assays were performed in a similar fashion but without coating the filters with Matrigel.

### DNA extraction and methylation data processing

Genomic DNA was isolated from tissue samples with Allprep DNA/RNA Kit (Qiagen) and arrayed using Infinium HumanMethylation450 BeadChips (450 K array, Illumina) to detect genome-wide methylation. We then conducted data preprocessing, normalization and calculation of β-value using the R package minfi^77^ (version 1.26.2). We applied the following criteria for quality control: (i) probes with detection *P* ≥ 0.01 in >5% of samples were removed from all samples; (ii) probes on the X or Y chromosome were removed; (iii) probes overlapping with single nucleotide polymorphisms (SNPs) were removed; (iv) probes mapped to multiple sites in human genome were removed. Finally, 429,717 probes were kept for further analysis.

The CpG probe annotation file was downloaded from the ENCODE Project database (http://genome.ucsc.edu/ENCODE/downloads.html). Each CpG probe is annotated with the corresponding gene, genomic region (TSS1500, 200–1500 bases upstream of the transcriptional start site [TSS]; TSS200, 0–200 bases upstream of the TSS; 5’UTR, within the 5’ untranslated region, between the TSS and the ATG start site; body, between the ATG and the stop codon; irrespective of the presence of introns, exons, TSS, or promoters; 3’UTR, between the stop codon and the poly A signal), the CpG island-associated regions (shore, 0–2 kb from island; shelf, 2–4 kb from island; N, upstream 5’ of CpG island; S, downstream 3’ of CpG island) and functional regions (enhancer, predicted enhancer elements as annotated in the original 450 K design are marked “true”; DHS, DNase I hypersensitivity site).^78^

### Identification of differentially methylated CpG sites

We applied a two-sided Wilcoxon signed-rank test to identify CpG sites (DMCs) differentially methylated between paired tumor and normal samples. *P* values were adjusted for multiple testing using the Benjamini–Hochberg method to control the false discovery rate (FDR). We required that significant DMCs have FDR *q* < 0.05 and the absolute median methylation difference (|MMD | ) > 0.20. We compared the of each DMC in ESCC and matched adjacent normal samples to determine its methylation status. A DMC in the ESCC genome of a specific patient is considered hyper-methylated or hypo-methylated if the β-value of this CpG site minus the β-value of the same site in that patient’s matched adjacent normal tissue sample is greater or less than 0.20, respectively. We counted the fraction of hyper-methylated or hypo-methylated CpG sites as the frequencies of hyper- or hypo-methylation events for each patient.

### Methylation quantitative trait loci (meQTL) analysis

The single nucleotide polymorphism (SNP) data of the 91 study subjects were obtained from our previous DNA sequencing study.^25^ From a total of 6,092,313 SNPs that have minor allele frequency ≥5% and no deviation from the Hardy-Weinberg equilibrium (*P* < 1.00e-6), we only selected SNPs within a 100-kb window centering each DMC on the same chromosome for meQTL mapping. An additive linear regression model implemented in the R package MatrixEQTL (v.2.3) was used and only SNPs with FDR *q* < 0.05 were deemed significant. A hypergeometric test was then used to assess the statistical significance of the overlap between identified meQTLs and potential ESCC risk SNPs obtained from the CCGD-ESCC database.^37^

### Identification of differentially expressed genes and gene set enrichment analysis

We identified genes differentially expressed between paired tumor and normal tissue samples using Student’s *t* test on log2-transformed gene expression levels (quantified by Transcript per Million, TPM). Only genes had FDR *q* < 0.05 and the relative fold change of mean expression levels > 2 or < 0.50 (tumor versus normal) were deemed significant. We replaced Student’s *t* test with Wilcoxon signed-rank test and 99.75% of the genes were still differentially expressed (FDR *q* < 0.05, relative fold change > 2 or < 0.50), including all the genes we used for downstream analyses. Gene ontology (GO) analysis was conducted using the enrichGO function implemented in the R package clusterProfiler (v. 3.8.1)^79^ and only the top 10 enriched GO terms were plotted.

### Identification of correlations between DNA methylation and gene expression

We examined the correlations between the methylation levels of DMCs and the expression levels of their corresponding genes using Spearman’s rank correlation and considered a correlation statistically significant if FDR *q* < 0.05 and the absolute Spearman rank correlation coefficient |*r* | > 0.30. We further considered the consistency of direction between the methylation level of DMC and the corresponding gene expression level for a more rigorous screening. For DMCs located in promoter, we applied the following criteria: (a) negative correlations (Spearman *r* < −0.30, *P* < 0.05) between DMCs and their corresponding genes; (b) hyper-methylation of DMC corresponding silencing of gene expression or hypo-methylation of DMC corresponding upregulation of gene expression. For DMCs located in gene-body, we applied the inverse criteria: (a) positive correlations (Spearman *r* > 0.30, *P* < 0.05) between DMCs and their corresponding genes; (b) hyper-methylation of DMC corresponding upregulation of gene expression or hypo-methylation of DMC corresponding silencing of gene expression.

### Development of a panel of DMCs for ESCC diagnosis

The panel was developed in 4 steps: (a) randomly divide 91 patients into the training (*n* = 60) and the validation (*n* = 31) sets with a 2:1 ratio; (b) From all the 1034 DMCs identified from 91 patients (method described above), select important variables for the training set using random forest analysis, with the feature dropping fraction of each iteration set at 1/3 according to the importance score; (c) use the least absolute shrinkage and selection operator (LASSO)-penalized logistic regression (a binomial model, 10-fold cross-validation) to further select the variables obtained in the previous step; (d) carry out the diagnostic model in the validation dataset and TCGA ESCC methylation dataset.

### Development of a pane of DMCs for prognostic risk prediction

For each DMC identified in our patient set, we fitted a univariate Cox proportional hazard model with that DMC as the covariate and only retained DMCs with nominal *P* < 0.05. Then, for each retained DMC, we fit a multivariate Cox proportional hazard model with that DMC as the predictor variable and age, sex, smoking status, drinking status and tumor TNM stage as covariates, and again, only retain DMCs with nominal *P* < 0.05. A sum of the methylation level of each remaining DMC multiplied by its respective natural logarithm of hazard ratio (HR) in our patient sample is the prognostic prediction model. And we applied the prognostic model in both our patients and TCGA ESCC patients.

### Other analyses

Unsupervised hierarchical clustering based on the methylation difference between ESCC and adjacent normal tissue samples was conducted using the pheatmap function implemented in the R package pheatmap (v. 1.0.12). The R package survival (v. 3.2-7) and survminer (v. 0.4.8) were used for survival analysis. Overall survival time was estimated by the Kaplan–Meier method and the differences were examined by the log-rank test. Hazard ratios (HRs) and their 95% confidence intervals (CIs) were calculated with the Cox proportional hazards model. All statistical tests were two-sided tests and *P* < 0.05 was considered significant unless indicated. We used R 3.6.1 (https://www.r-project.org/).

## Supplementary information

Xi et al_Supplementary Information

Xi et al_Supplementary Data

## Data Availability

The methylation data generated in this study are deposited in the OMIX, China National Center for Bioinformation/Beijing Institute of Genomics, Chinese Academy of Sciences (http://bigd.big.ac.cn/omix, accession number OMIX267). The other genetic and transcriptomic data of the same individuals are available through our earlier publications.^25^ We obtained DNA methylation data (level 3) of 95 ESCCs and 14 normal esophageal tissue samples from The Cancer Genome Atlas (TCGA) (http://gdac.broadinstitute.org/) for comparative analysis. We also obtained methylation data from the Gene Expression Omnibus (GEO) database (GSE52826 and GSE77991), which include 4 ESCCs, 14 normal tissues adjacent to tumors from patients, and 21 esophageal mucosal tissues from healthy individuals. Methylation data of 16 ESCC markers in TCGA 22 cancer types were downloaded from SMART (Shiny methylation analysis resource tool) app.^80^

## Code availability

We used published software for all our analyses as indicated. Other accompanying code is available from the authors upon request.
