# Supplementary material for: Multi-omic characterization of genome-wide abnormal DNA methylation reveals diagnostic and prognostic markers for esophageal squamous-cell carcinoma
Source: Signal Transduct Target Ther. 2022 Feb 25;7:53. doi: 10.1038/s41392-022-00873-8 (PMC8873499; doi:10.1038/s41392-022-00873-8)
Supplement: Supplementary file 1 — Xi et al_Supplementary Information [file 41392_2022_873_MOESM1_ESM.docx]

Supplementary Information

**Multi-omic characterization of genome-wide abnormal DNA methylation reveals diagnostic and prognostic markers for esophageal squamous-cell carcinoma**

Yiyi Xi, Yuan Lin, Wenjia Guo, Xinyu Wang, Hengqiang Zhao, Chuanwang Miao, Weiling, Liu, Yachen Liu, Tianyuan Liu, Yingying Luo, Wenyi Fan, Ai Lin, Yamei Chen, Yanxia Sun, Yulin Ma, Xiangjie Niu, Ce Zhong, Wen Tan, Meng Zhou, Jianzhong Su*, Chen Wu*, Dongxin Lin*

*Correspondence:

Chen Wu (chenwu@cicams.ac.cn)

Dongxin Lin (lindx@cicams.ac.cn)

Jianzhong Su (sujz@wmu.edu.cn)

This file includes:

Supplementary Figures S1 to S10

Supplementary Table S1 to S4

**Supplementary Fig. S1. The genomic distribution of differentially methylated CpG sites in ESCC.**

**a** and **b.** The proportion (**a**) and odds ratio (**b**) of differentially methylated CpG sites (DMCs) in different chromosomes. **c** and **d**. The proportions (**c**) and odds ratio (**d**) of DMCs in various genomic regions. **e**−**j**. The proportions and odds ratio of DMCs in Chromosomes 8 (**e** and **f**), 18 (**g** and **h**) and 19 (**i** and **j**) in various genomic regions. Island, CpG island; shore, 0−2 kb from CpG island; shelf, 2−4 kb from CpG island; open sea, other genomic regions; TSS1500, 200−1500 bases upstream of the transcriptional start site (TSS); TSS200, 0−200 bases upstream of the TSS; 5'UTR: within the 5'untranslated region (UTR), between the TSS and the ATG start site; body, between the ATG and stop codon regardless the presence of introns, exons, TSS, or promoters; 3'UTR: between the stop codon and poly A signal. The odds ratio was computed against the general distribution and the *P* value was computed by Hypergeometric test using the R function ‘phyper’.

**
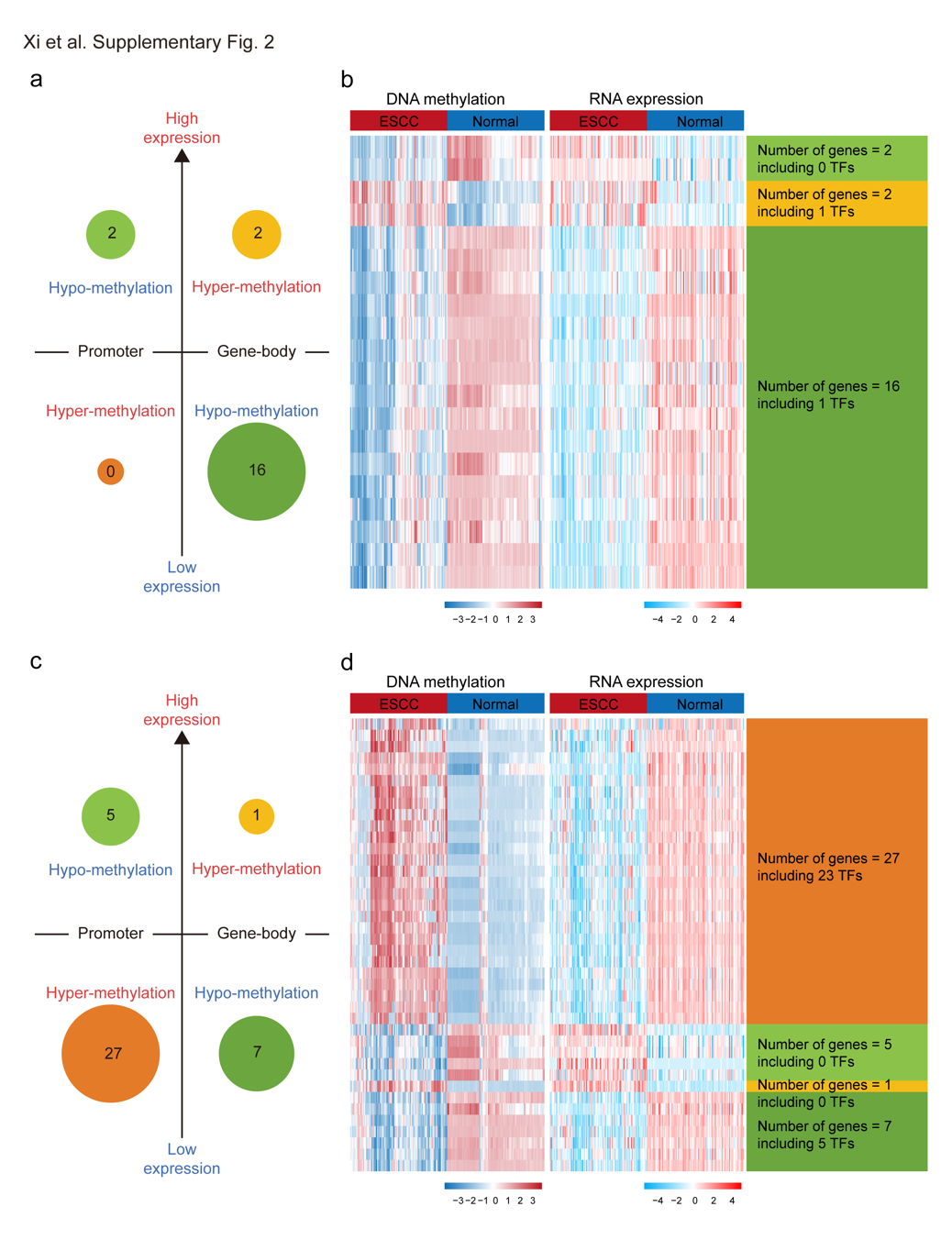
**

**Supplementary Fig. S2. Integrative analysis of whole-genome DNA and RNA-sequencing data uncovered methylation-expression correlations in Chromosomes 8 and 19.**

The association between promoter or gene-body methylation and host gene expression were identified in Chromosomes 8 (**a** and **b**) and 19 (**c** and **d**). There are four clusters: genes (n = 0, n = 27) that are hyper-methylated in promoter with low expression in ESCC; genes (n = 2, n =5) that are hypo-methylated in promoter with high expression; genes (n = 2, n =1) that are hyper-methylated in gene-body with high expression; genes (n = 16, n =7) that are hypo-methylated in gene-body with low expression, respectively for Chromosomes 8 and 19. Number of known TFs are shown in each cluster.


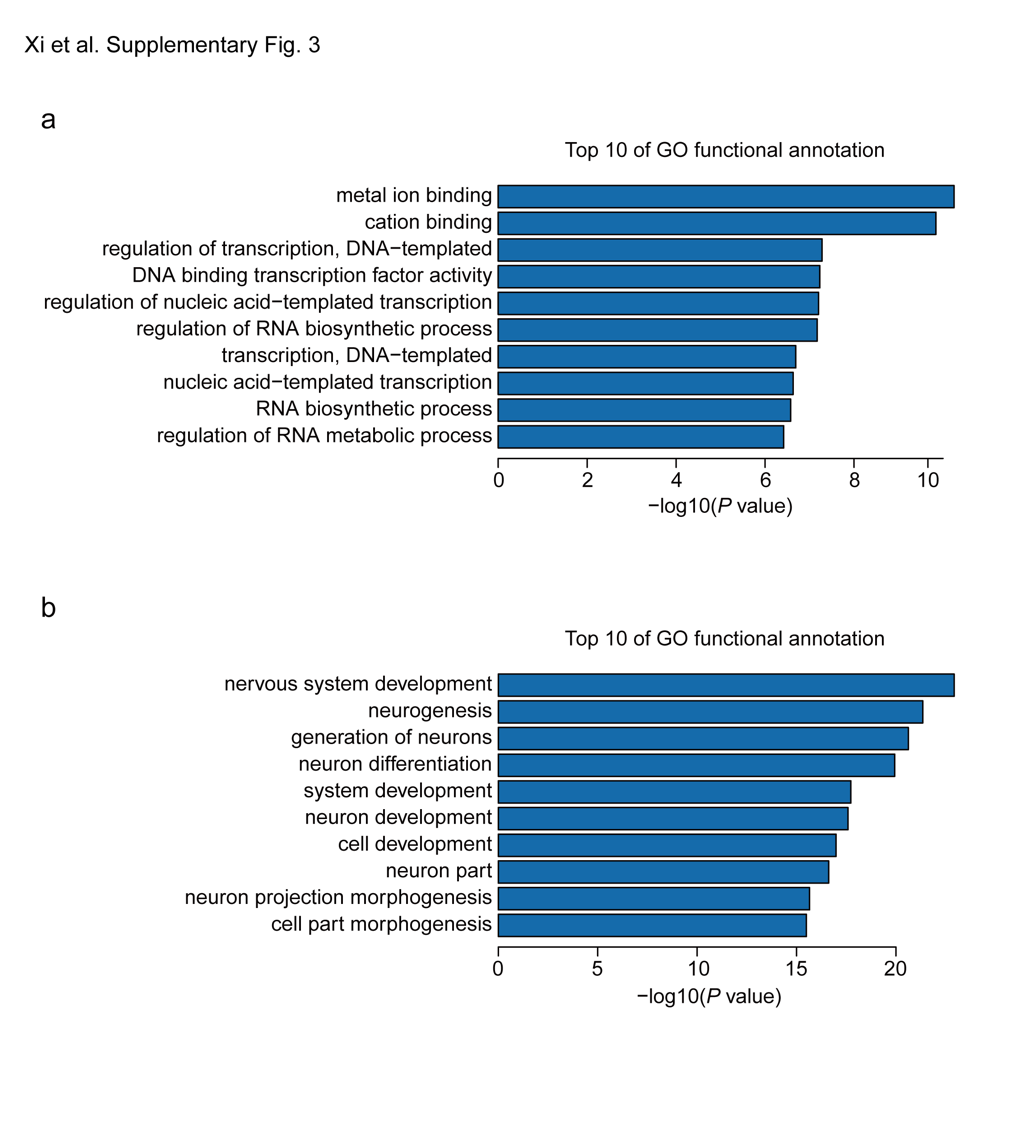


**Supplementary Fig. S3.** **Functional annotation of the genes with aberrant DNA methylation.**

**a** and **b.** Gene ontology (GO) functional annotation of negatively (**a**) or positively (**b**) correlated genes. The top 10 categories are shown for each group, ranked by the log10-transformed *P* value.


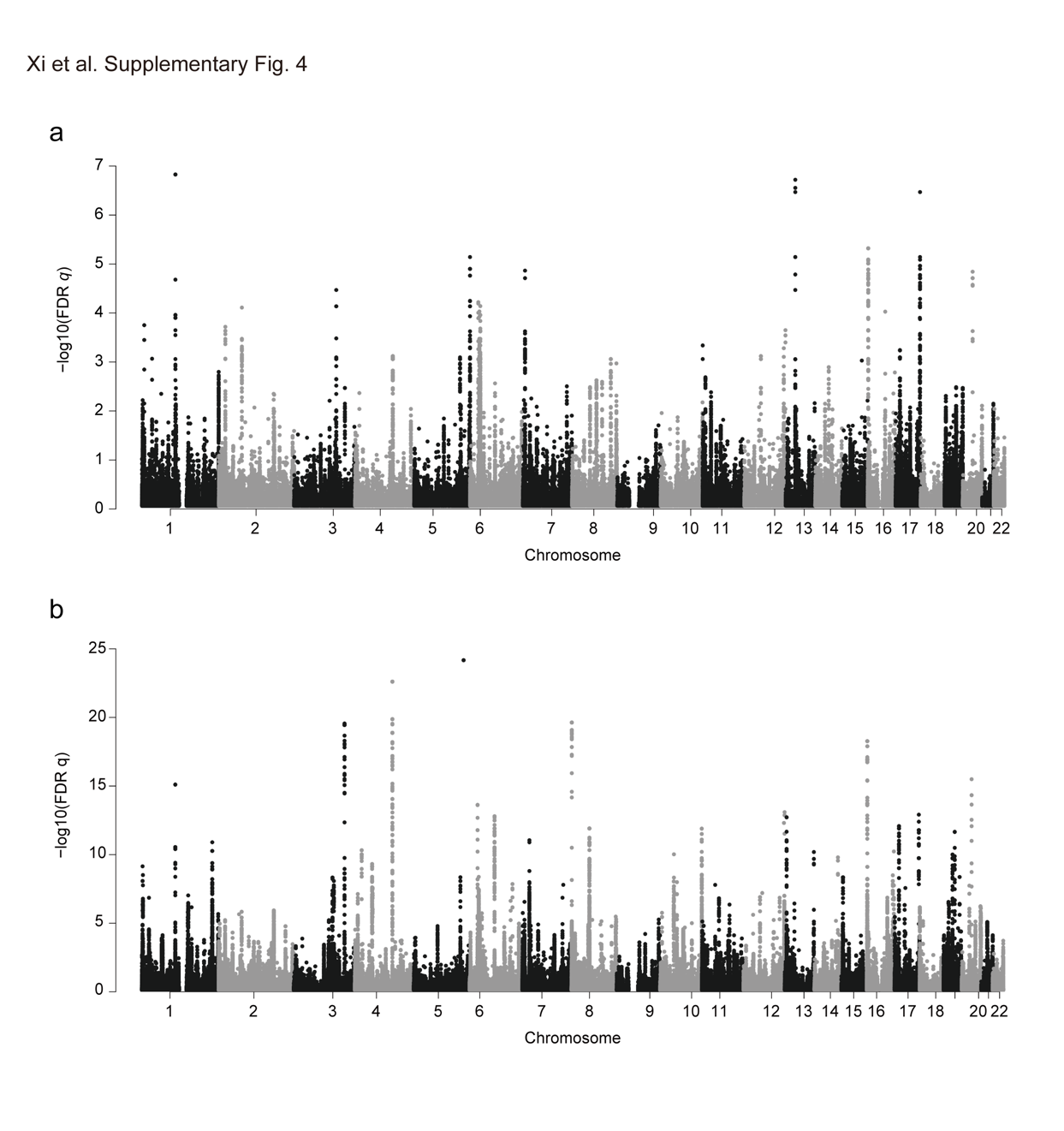


**Supplementary Fig. S4. Genome-wide identification of *Cis*-meQTLs in all DMC sites.**

Manhattan plots of all the *Cis*-meQTLs (i.e., SNPs) in ESCC (a) and adjacent normal tissue samples (b). The x-axis represents the genomic position of each meQTL and the y-axis represents the -log10-transformed FDR *q* value of each SNP.


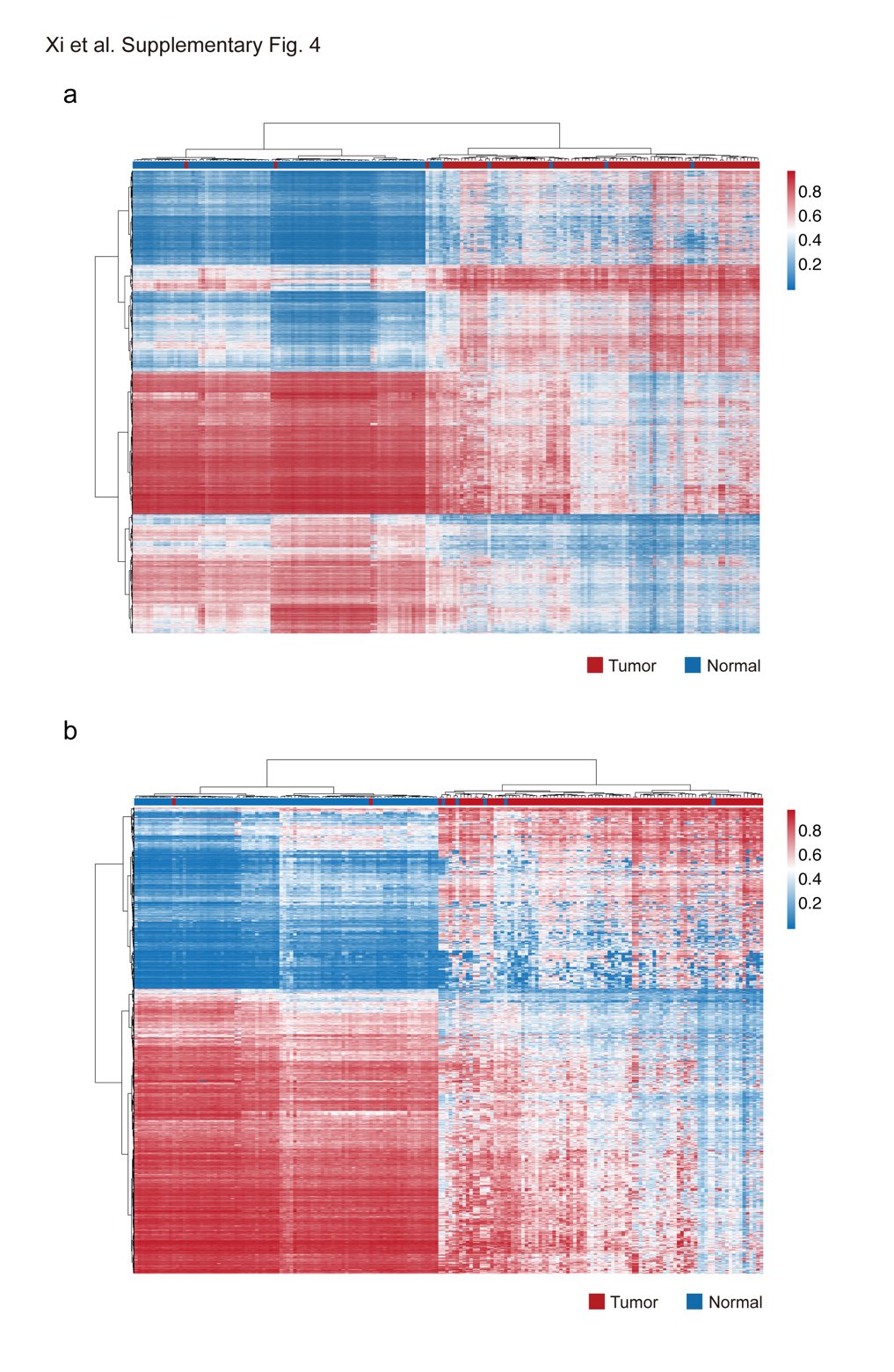


**Supplementary Fig. S5.** **Unsupervised hierarchical clustering and heatmap in training sample set of differentially methylated CpG sites with potential function.**

**a**. Unsupervised hierarchical clustering of DMCs and heatmap for methylation profiles of 91 pairs of tissues. Each column represents a sample; each row represents a CpG site. The scale represents the methylation beta values. Top color bars mark the tissue types. **b**. Unsupervised hierarchical clustering of the 1,034 sites associated with their host genes expression levels in training set. Each column represents a sample, and each row represents a CpG site. The scale represents the methylation beta values. Top color bars mark the type of tissue sample.


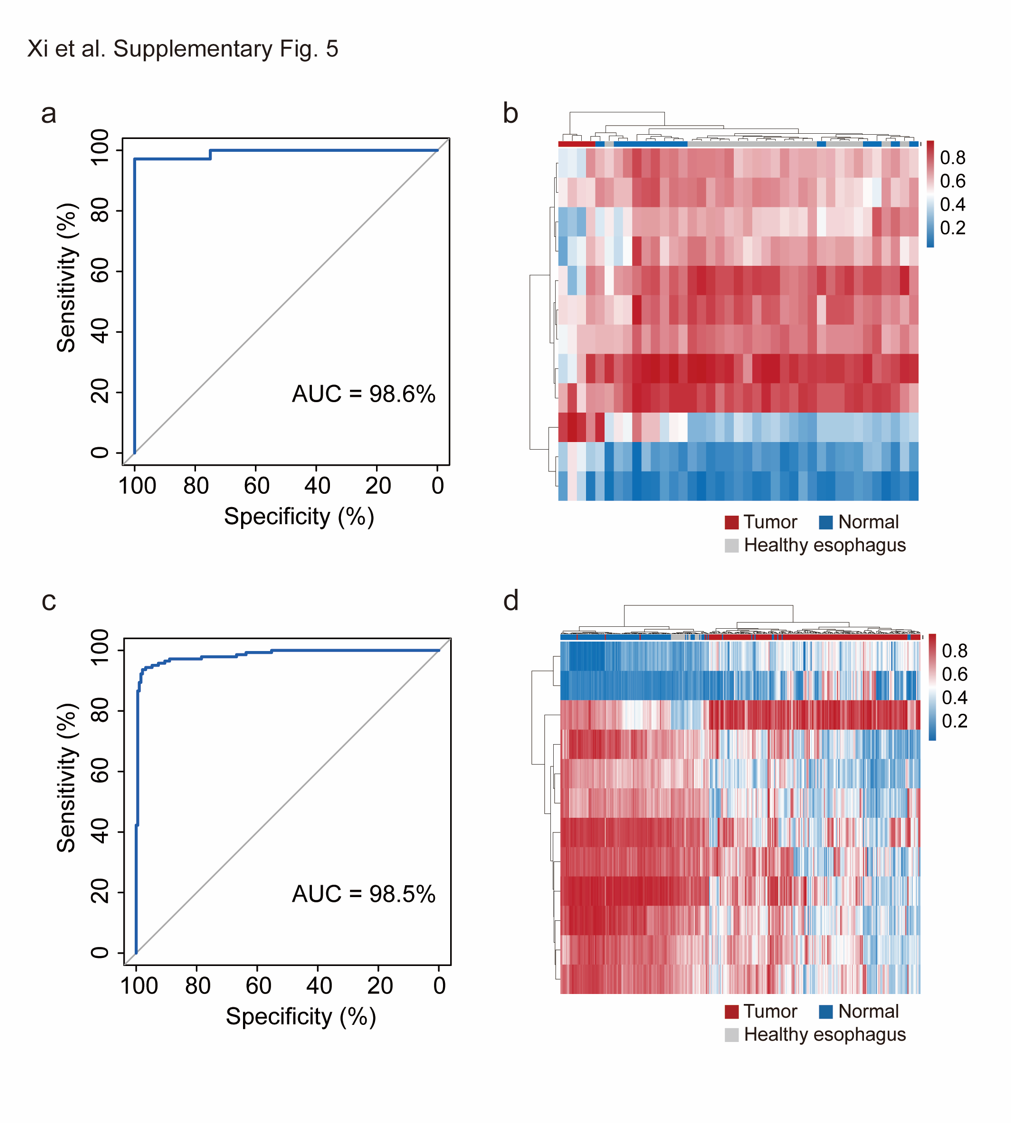


**Supplementary Fig. S6. Validation of the probes-based diagnostic panel in GEO and the entire dataset.**

**a** and **b.** The receiver operation curve (ROC) of the diagnostic panel (**a**) and unsupervised hierarchical clustering and heatmap of the 12 methylation markers in panel (**b**) in the Gene Expression Omnibus (GEO) dataset. **c** and **d**. The ROC of the diagnostic panel (**c**) and unsupervised hierarchical clustering and heatmap of the 12 methylation markers (**d**) in the entire dataset.

**
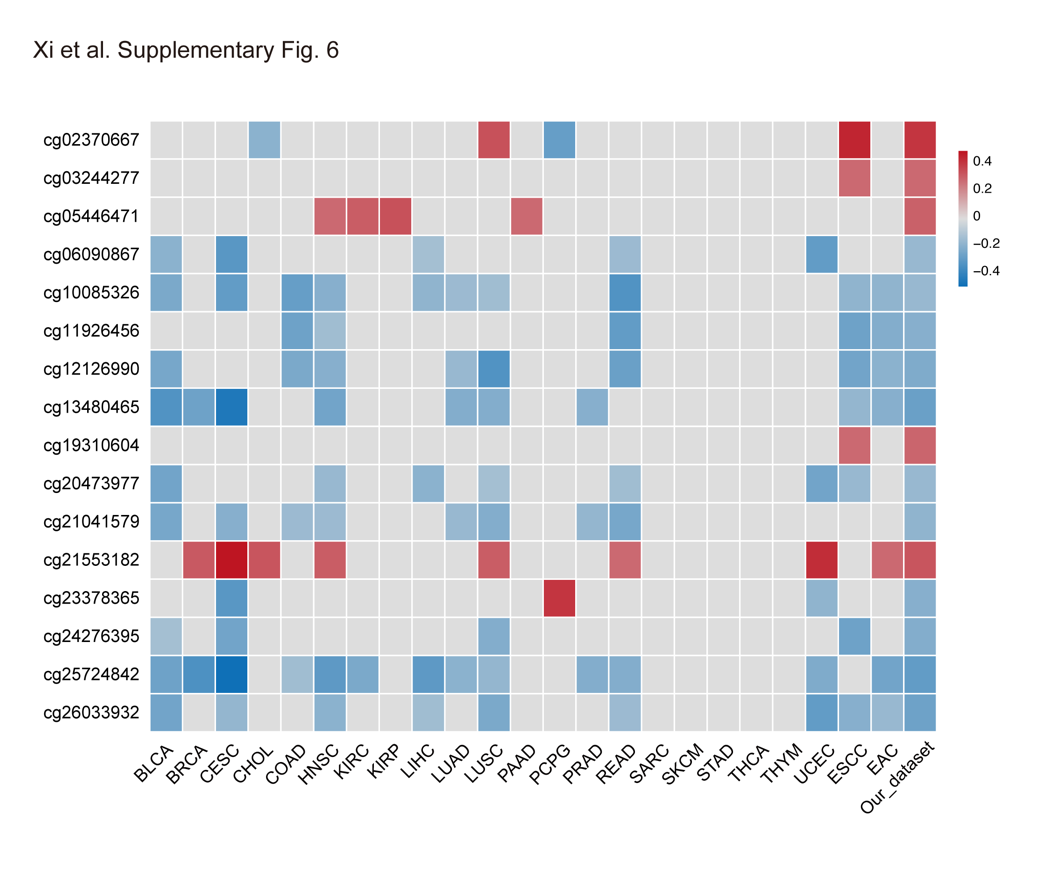
**

**Supplementary Fig. S7. Methylation patterns of 16 ESCC markers in 22 cancer types of TCGA.**

Heatmap for methylation profiles of 16 markers in two panels across 22 cancer types in TCGA database. Each column represents a cancer type; each row represents a marker. The scale represents the corresponding marker’s β-values difference between tumor and adjacent normal samples of the corresponding cancer type.


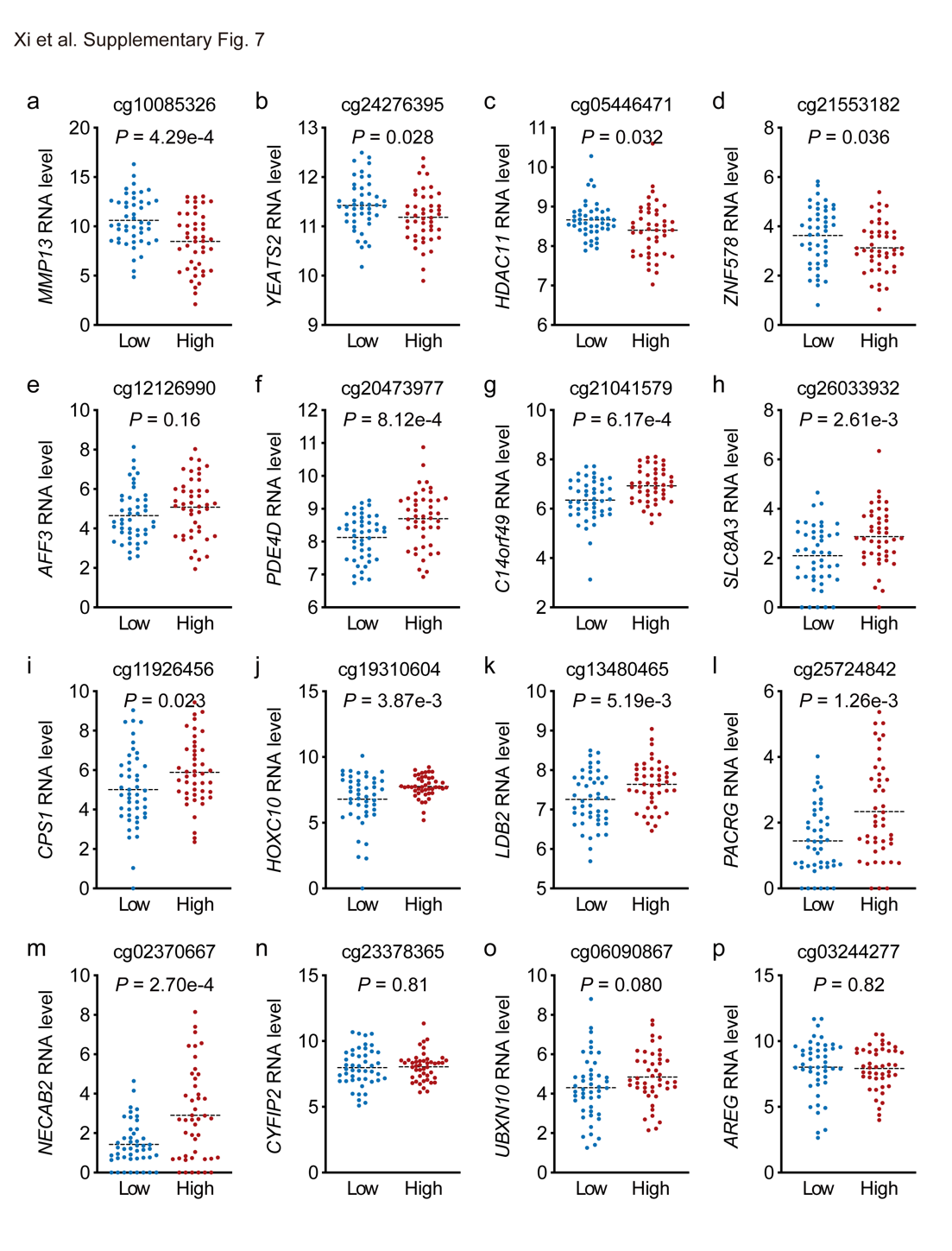


**Supplementary Fig. S8. Expression levels of the corresponding genes in low or high methylation group of each marker.**

**a−l.** The expression level of corresponding gene in low- or high-methylation group of each marker in the diagnostic panel. **m−p.** The expression level of corresponding gene in low- or high-methylation group of each marker in the prognostic panel. Patients were classified into low- or high-methylation group based on the median methylation level of each marker. Gene mRNA expression level (TPM) was added by 1 and then log2 transformed. Dotted short line indicates mean expression level of each gene. The significance of difference between low- and high-methylation groups was examined by Student’s *t*-test.

**
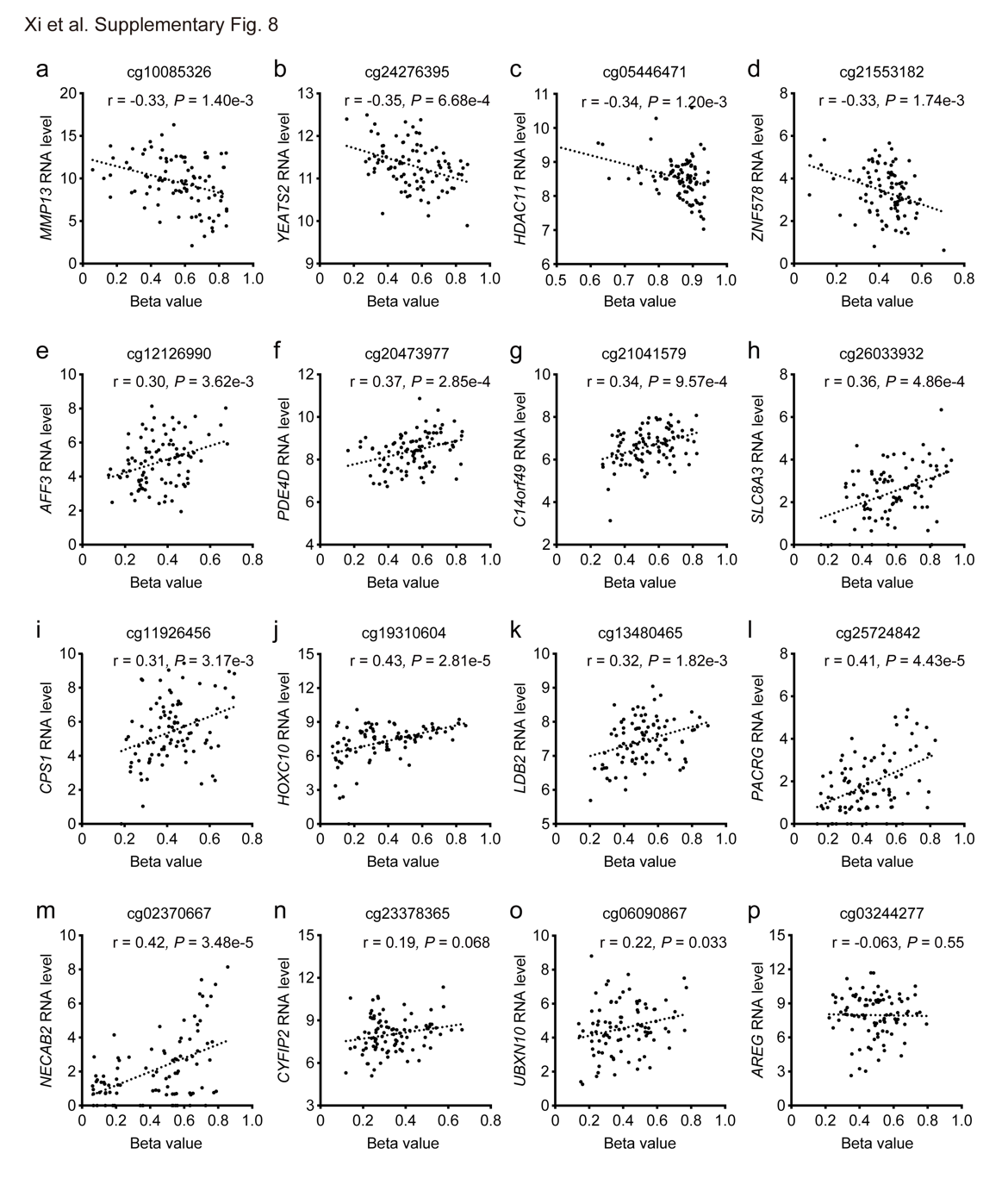
**

**Supplementary Fig. S9. The Spearman correlation between the levels of methylation and mRNA expression of genes in diagnostic and prognostic panels.**

The *y* axis represents the gene expression level, and the *x* axis represents beta value of methylation. Gene mRNA expression level (TPM) was added by 1 and then log2 transformed.

**
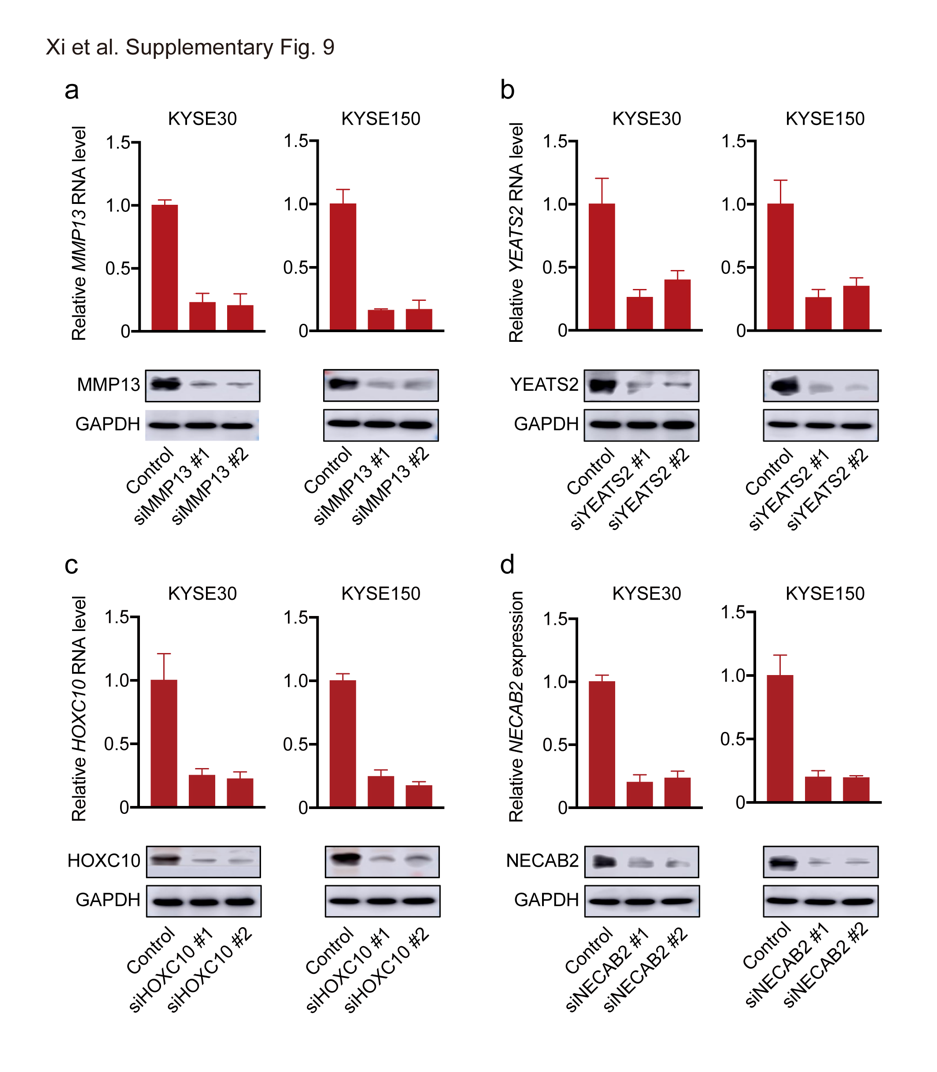
**

**Supplementary Fig. S10. The knockdown efficiency of marker genes in ESCC cells.**

The mRNA expression levels and protein levels of *MMP13* (**a**), *YEATS2* (**b**), *HOXC10* (**c**) and *NECAB2* (**d**) subjected to siRNA silencing in KYSE30 and KYSE150 cells, respectively. Data represent mean ± SEM from 3 independent experiments and each had three replications.

**Supplementary Table S1.** Characteristics of 12 methylation markers in the diagnostic panel

| Marker | Chr.* | Genomic position | Host gene | Location | |
| --- | --- | --- | --- | --- | --- |
| cg05446471 | 3 | 13522740 | *HDAC11* | 5'UTR | |
| cg10085326 | 11 | 102826680 | *MMP13* | TSS1500 | |
| cg11926456 | 2 | 211432972 | *CPS1* | Gene-body^#^ | |
| cg12126990 | 2 | 100588044 | *AFF3* | Gene-body^#^ | |
| cg13480465 | 4 | 16795757 | *LDB2* | Gene-body^#^ | |
| cg19310604 | 12 | 54383389 | *HOXC10* | 3'UTR | |
| cg20473977 | 5 | 59193490 | *PDE4D* | Gene-body | |
| cg21041579 | 14 | 95907997 | *SYNE3* | Gene-body^#^ | |
| cg21553182 | 19 | 52956821 | *ZNF578* | TSS200^+^ | |
| cg24276395 | 3 | 183419891 | *YEATS2* | 5'UTR | |
| cg25724842 | 6 | 163574564 | *PACRG* | Gene-body | |
| cg26033932 | 14 | 70547465 | *SLC8A3* | Gene-body | |
| *Chr., chromosome; UTR, untranslational region; TSS, translation start site.  ^#^Located in the predicted enhancer element.  ^+^Located in DNase I hypersensitivity site. | | | | |  |

**Supplementary Table S2.** Multivariate Cox regression analysis of 4 methylation markers

| Marker | HR* | 95% CI* | *P* value |
| --- | --- | --- | --- |
| Our ESCC dataset | | | |
| cg02370667 | 2.18 | 1.26 - 3.78 | 3.40e-3 |
| cg23378365 | 1.80 | 1.04 - 3.12 | 8.48e-4 |
| cg06090867 | 1.76 | 1.03 - 3.00 | 0.043 |
| cg03244277 | 2.27 | 1.33 - 3.87 | 6.73e-4 |
| TCGA-ESCC dataset | | | |
| cg02370667 | 4.71 | 1.77 - 12.55 | 1.94e-3 |
| cg23378365 | 1.86 | 0.76 - 4.57 | 0.17 |
| cg06090867 | 3.43 | 1.32 - 8.94 | 0.012 |
| cg03244277 | 2.49 | 1.01 - 6.15 | 0.049 |
| HR (hazard ratio) and 95% CI (confidence interval) in our ESCC dataset was computed with Cox hazard proportion model adjusted for age, sex, smoking status, drinking status and tumor TNM stage; in TCGA-ESCC dataset was adjusted only for available factors including age, sex and tumor TNM stage. | | | |

**Supplementary Table S3.** Characteristics of the 4 methylation markers in the prognostic panel

| Marker | Chr.* | Genomic position | Host gene | Location |
| --- | --- | --- | --- | --- |
| cg02370667 | 16 | 84029511 | *NECAB2* | Gene-body^#^ |
| cg23378365 | 5 | 156696351 | *CYFIP2* | TSS200 |
| cg06090867 | 1 | 20511782 | *UBXN10* | TSS1500 |
| cg03244277 | 4 | 75310450 | *AREG* | TSS1500 |
| *Chr., chromosome; TSS, translation start site.  ^#^Located in the predicted enhancer element and DNase I hypersensitivity site. | | | | |

**Supplementary Table S4.** Small interfering RNA sequences and qualitative PCR (qPCR) primer sequences for the genes interested

| Gene | |  | Small interfering RNA sequence (5'→3') | |  |
| --- | --- | --- | --- | --- | --- |
| *MMP13* | | #1 | GCUGUUCACUUUGAGGAUATT | |  |
|  | |  | UAUCCUCAAAGUGAACAGCTT | |  |
|  | | #2 | GCAUCUGGAGUAACCGUAUTT | |  |
|  | |  | AUACGGUUACUCCAGAUGCTT | |  |
| *YEATS2* | | #1 | CCUUCAUCCUAGCUAUAAATT | |  |
|  | |  | UUUAUAGCUAGGAUGAAGGTT | |  |
|  | | #2 | GCGGAUAGAUAUCAUACAUTT | |  |
|  | |  | AUGUAUGAUAUCUAUCCGCTT | |  |
| *HOXC10* | | #1 | ACCUAGUGUCAAGGAGGAGAATT | |  |
|  | |  | UUCUCCUCCUUGACACUAGGUTT | |  |
|  | | #2 | CUUACAGACAGACAAGUCAAATT | |  |
|  | |  | UUUGACUUGUCUGUCUGUAAGTT | |  |
| *NECAB2* | | #1 | CCUGAAGGCCAUGGGUUAUTT | |  |
|  | |  | AUAACCCAUGGCCUUCAGGTT | |  |
|  | | #2 | GGUUAUACCAAGAAGGUAUTT | |  |
|  | |  | AUACCUUCUUGGUAUAACCTT | |  |
| Gene | |  | qPCR primer sequence (5'→3') | |  |
| *MMP13* | Forward | | | CCTTGATGCCATTACCAGTCTCC | |
|  | Reverse | | | AAACAGCTCCGCATCAACCTGC | |
| *YEATS2* | Forward | | | TTACTGCCGCTGGTCCTCAGAA | |
|  | Reverse | | | AGAGTTTAGTGCCAGGAGGCAAA | |
| *HOXC10* | Forward | | | GAGCGAAAAGGAGAGGGCCAAA | |
|  | Reverse | | | TCCGCTCTTTGCTGTCAGCCAA | |
| *NECAB2* | Forward | | | TGAGGCTCTCAGATGGCTTCAC | |
|  | Reverse | | | TGGCTCAGTGTGTCCACCTTGA | |
